# Vickers Micro-Hardness of New Restorative CAD/CAM Dental Materials: Evaluation and Comparison after Exposure to Acidic Drink

**DOI:** 10.3390/ma12081246

**Published:** 2019-04-16

**Authors:** Marco Colombo, Claudio Poggio, Alessandro Lasagna, Marco Chiesa, Andrea Scribante

**Affiliations:** 1 Unit of Restorative Dentistry, Section of Dentistry, Department of Clinical, Surgical, Diagnostic and Paediatric Sciences, University of Pavia, 27100 Pavia, Italy; marco.colombo@unipv.it (M.Co.); claudio.poggio@unipv.it (C.P.); alessandro.lasagna01@universitadipavia.it (A.L.); marco.chiesa@unipv.it (M.Ch.); 2 Unit of Orthodontics and Paediatric Dentistry, Section of Dentistry, Department of Clinical, Surgical, Diagnostic and Paediatric Sciences, University of Pavia, 27100 Pavia, Italy

**Keywords:** CAD/CAM, dentistry, composite resins, ceramic, acidic drink, Vickers micro-hardness

## Abstract

CAD/CAM (computer-aided design/computer-aided manufacturing) for indirect restorative materials has been recently introduced in dentistry. The purpose of this study was to evaluate the change of the surface micro-hardness of different restorative CAD/CAM materials after exposure to a carbonated acidic drink (Coca-Cola, Coca-Cola Company, Milan, Italy). One hundred and eighty specimens of identical size (2 mm thickness) were obtained by sectioning each tested CAD/CAM block of four materials: a hybrid ceramic (CERASMART™, GC Corporation, Tokyo, Japan), a resin nano ceramic (Lava™ Ultimate, 3M, Monrovia, CA, USA), a nanohybrid composite (Grandio blocs, VOCO GmbH, Cuxhaven, Germany), and a zirconia-reinforced lithium silicate glass ceramic (VITA SUPRINITY^®^ PC; VITA Zahnfabrik, Bad Sackingen, Germany). Forty-five specimens of each material were tested. Micro-hardness was measured at baseline, after 7 days and after 28 days. The data were analyzed. The micro-hardness of each material varied significantly after immersion in Coca-Cola. The nanohybrid composite had a high initial micro-hardness and the greatest percentage loss after acid exposure. The hybrid ceramic and the resin nano ceramic had similar percentage losses of micro-hardness values even if the second material had higher initial values. The zirconia-reinforced lithium silicate glass ceramic had the highest baseline values and the lowest percentage loss of micro-hardness. The different CAD/CAM materials presented different micro-hardness values before and after acid exposure.

## 1. Introduction

The consumption of energy drinks and carbonated drinks is very common, particularly in the age range of 18–35 years. Many studies demonstrated that their consumption can cause dental erosion and consequently restoration erosion [1,2]. The presence of some acids, including organic acids such as acetic, propionic, and lactic acid in the formulation of beverages can lead to a decrease in the micro-hardness values of resin composites [3,4]. A more controlled consumption of soft drinks has a positive influence on diet and on dental and dental materials erosion. Coca-Cola is a widely consumed carbonated beverage, with a pH level of 2.52. 

CAD/CAM (computer-aided design/computer-aided manufacturing) technology was introduced in dentistry during the 1980s, originally called CEREC because it was used to mill ceramic materials (CEramic REConstruction) [5]. This technology is nowadays widely used in the production of frameworks in conservative dentistry [6], prosthodontics [7], orthodontics [8], and pediatric dentistry [9].

Some authors claimed that the production and application of restorations prepared with CAD/CAM technology systems provide better performances than restorations performed with conventional laboratory procedures in terms of esthetic quality, clinical duration, and marginal precision [10,11]. Moreover, patients also reported positive feedback regarding full digital CAD/CAM workflow [12].

When selecting a restorative material, one of the main considerations is its mechanical properties. As a restorative material is used to replace missing tooth structure, it needs to be strong enough to withstand the forces associated with mastication [13]. A micro-hardness test can be used to evaluate these mechanical properties. The micro-hardness is strictly related to the composition characteristics of the materials tested, and it is influenced by aging, water absorption, and reactions of the material surface [14]. Micro-hardness measurements are conducted using indentation tests (with Vickers or Knoop indenters), which can give a good determination of the resistance to localized plastic deformation [15]. This aspect has great importance in dentistry as surface behavior is related to usury and scratch, thus linking resistance to clinical long-term efficiency [16].

Previous studies on CAD/CAM frameworks evaluated micro-hardness [17], flexural characteristics [18], fatigue behavior [19], bond strength [20], and chipping resistance [21]. To our knowledge, there are no studies that evaluated the micro-hardness of CAD/CAM materials after exposure to acidic drink. Therefore, the purpose of the present report was to measure and compare the micro-hardness of four different newly introduced CAD/CAM materials.

## 2. Materials and Methods

Four types of restorative CAD/CAM materials were used in this in vitro study: a force-absorbing hybrid ceramic block (CERASMART™, GC Corporation, Tokyo, Japan), a resin nano ceramic (Lava™ Ultimate, 3M, Monrovia, CA, USA), a nanohybrid composite (Grandio blocs, VOCO GmbH, Cuxhaven, Germany), and a zirconia-reinforced lithium silicate glass ceramic (VITA SUPRINITY^®^ PC, VITA Zahnfabrik, Bad Sackingen, Germany). The specifications of the materials tested are listed in Table 1. For each brand, the A2 Vita shade was selected.

In the present in vitro study, 45 specimens of each of the four materials were prepared. For each restorative material, the CAD/CAM block was sectioned to obtain specimens of identical size (2 mm thickness) with a G5 milling machine (Dental Machine Srl, Bobbio PC, Emilia-Romagna, Italy) and the related software (WX Prop View; Matrix Vision GmbH, Oppenweiler Germany).

Before testing their properties, VITA SUPRINITY^®^ PC was crystallized with VITA VACUMAT 600 M (VITA Zahnfabrik, Bad Sackingen, Germany) and each specimen was polished with super fine (600 P) and ultra-fine (1200 P and 1500 P) abrasive sandpaper (SandBlaster, 3M, Monrovia, CA, USA). The polishing procedures were conducted under dry conditions for 30 s of each abrasive sandpaper and before the next polishing step each specimen was rinsed with water and allowed to air-dry [1].

For each restorative CAD/CAM material, the 45 specimens were randomly divided into three groups (*n* = 15 per group). The micro-hardness of each CAD/CAM material was tested at baseline (T0), after immersion in a sealed vial containing 50 mL of acidic drink (Coca-Cola Company, Milan, Italy; pH 2.52) for 7 days (T1, *n* = 15) and after immersion in 50 mL of acidic drink (T2, *n* = 15). The specimens tested at T2 were rinsed with water weekly in order to prevent bacterial adhesion and the solution was changed to maintain the low pH level [22].

As in previous reports that evaluated the micro-hardness of dental materials [1,22], each specimen’s micro-hardness was determined with a micro-hardness tester (Isoscan HV2, LTF Spa, Antegnate, Bergamo, Italy) using a Vickers diamond indenter. Three indentations were made equally placed over a circle, each being no closer than 0.5 mm to the adjacent indentation (UNI EN ISO 6507 [23]), using a 1000 g load with a 15 s dwell time. The two diagonal lengths of each indentation were measured by a 40× magnification built-in scale microscope, and were converted into a micro-hardness value (VHN) using the following equation: HV = 1.854 P/d^2^, where HV is micro-hardness in kg/mm^2^, P is the load in kgf and d is the average length of the diagonals in mm. For a given specimen, the three hardness values for each surface were averaged and reported as a single value. Scanning electron microphotographs were taken before and after the indentation test for each group tested (mvBlueFOX3, MATRIX VISION Italia, Brescia, Italia). In the photomicrographs, the different surfaces of the four materials tested were evaluated before and after indentation at the three different immersion protocols (T0: no immersion; T1: 7 days; T2: 28 days). The micro-hardness values were analyzed using Stata 12 software (Stata, College Station, TX, USA). Descriptive statistics including the mean, standard error of mean, median, minimum, and maximum values were calculated for all groups. Statistical analysis of the results of micro-hardness testing [1,14,15] included the Shapiro–Wilk test to assess the normality of the distributions and the nonparametric Kruskal–Wallis analysis of variance (ANOVA) to assess the differences caused by the immersion protocol in the acidic drink for each material. A significant level of α = 0.05 was set for comparison between the groups. The mean percentage of micro-hardness loss after the immersion protocols was calculated for each CAD/CAM material and the groups were compared with a Tukey test (α = 0.05).

## 3. Results

The micro-hardness values of the four materials involved in the study are reported in Table 2. Descriptive statistics refer to the micro-hardness values of the three different conditions tested (T0, T1, and T2).

In the control group (T0), the values (731.4 ± 2.63) reported for the zirconia-reinforced lithium silicate glass ceramic (VITA SUPRINITY^®^ PC) were significantly higher than those of the other materials (*p* < 0.001), while the significantly lowest micro-hardness (68.22 ± 0.52) was measured for the hybrid ceramic block (CERASMART™) (*p* < 0.001).

**Figure 1:**
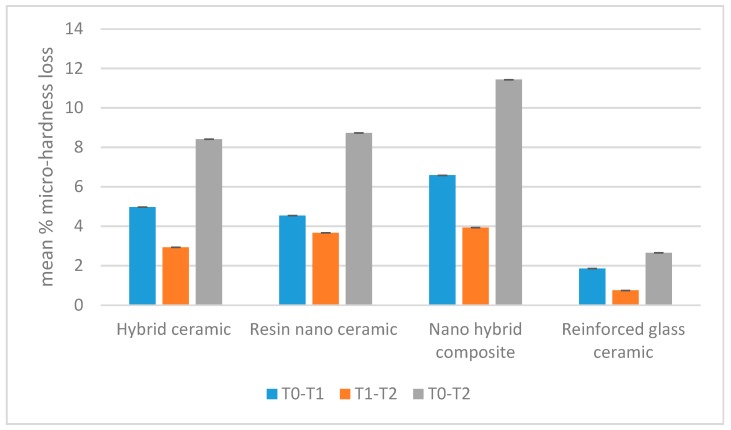
Mean percentage micro-hardness loss for different immersion protocols.

The ANOVA test within experimental replications showed that each tested material varied significantly after immersion in the acidic drink (*p* < 0.05). The main mean percentage micro-hardness loss was recorded after 7 days of immersion, while during the following 21 days the percentage loss was significantly lower (*p* < 0.05), as displayed in Figure 1 and reported in Table 3.

After 28 days of immersion (T2), the mean percentage of micro-hardness loss was not significantly different between the hybrid ceramic block (CERASMART™) (8.41%) and the resin nano ceramic (Lava™ Ultimate) (8.73%) (*p* > 0.05). The nanohybrid composite (Grandio blocs) showed the highest percentage in micro-hardness loss (13.4%) (*p* < 0.05). The zirconia-reinforced lithium silicate glass ceramic (VITA SUPRINITY^®^ PC) showed the lowest mean percentage (2.65%) (*p* < 0.05).

## 4. Discussion

**Figure 2:**
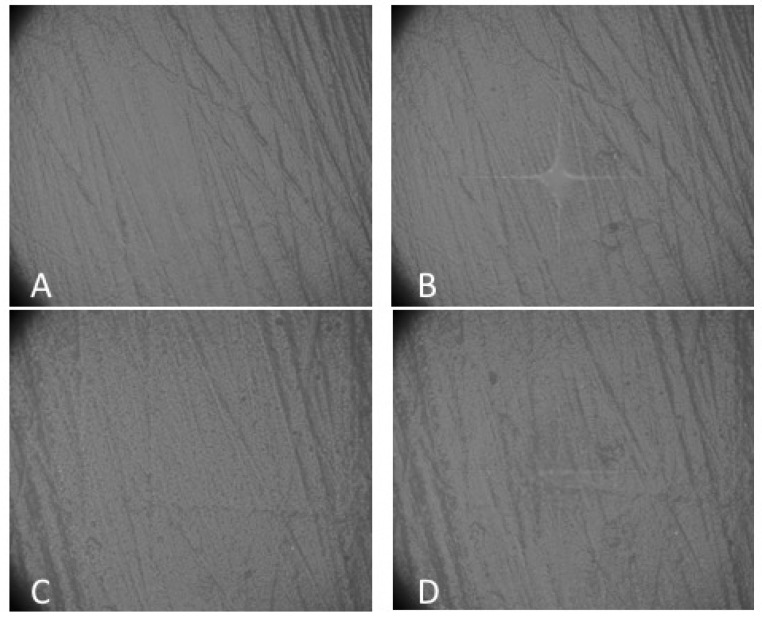

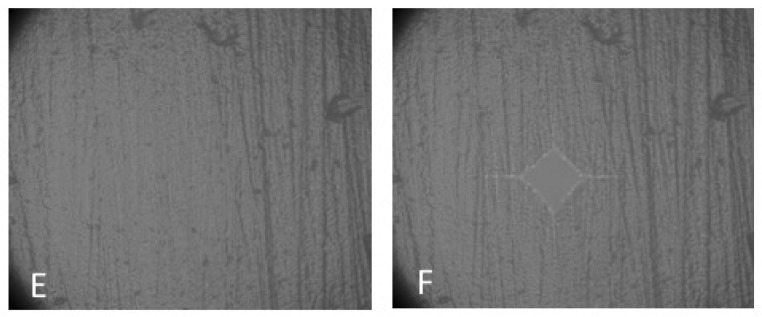
Photomicrographs of hybrid ceramic (CERASMART™) before and after immersion in acidic drink for 7 and 28 days (40× magnification). (**A**,**B**) Before immersion; (**C**,**D**) after immersion in acidic drink for 7 days, (**E**,**F**) after immersion in acidic drink for 28 days.

**Figure 3:**
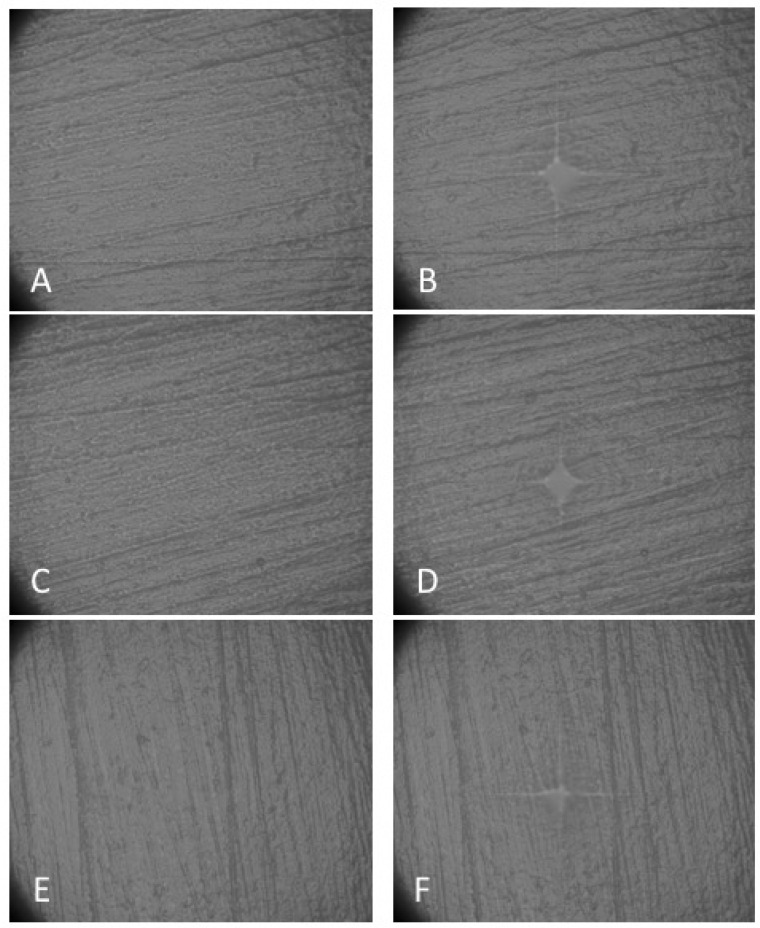
Photomicrographs of resin nano ceramic (Lava™ Ultimate) before and after immersion in acidic drink for 7 and 28 days (40× magnification). (**A**,**B**) Before immersion; (**C**,**D**) after immersion in acidic drink for 7 days, (**E**,**F**) after immersion in acidic drink for 28 days.

**Figure 4:**
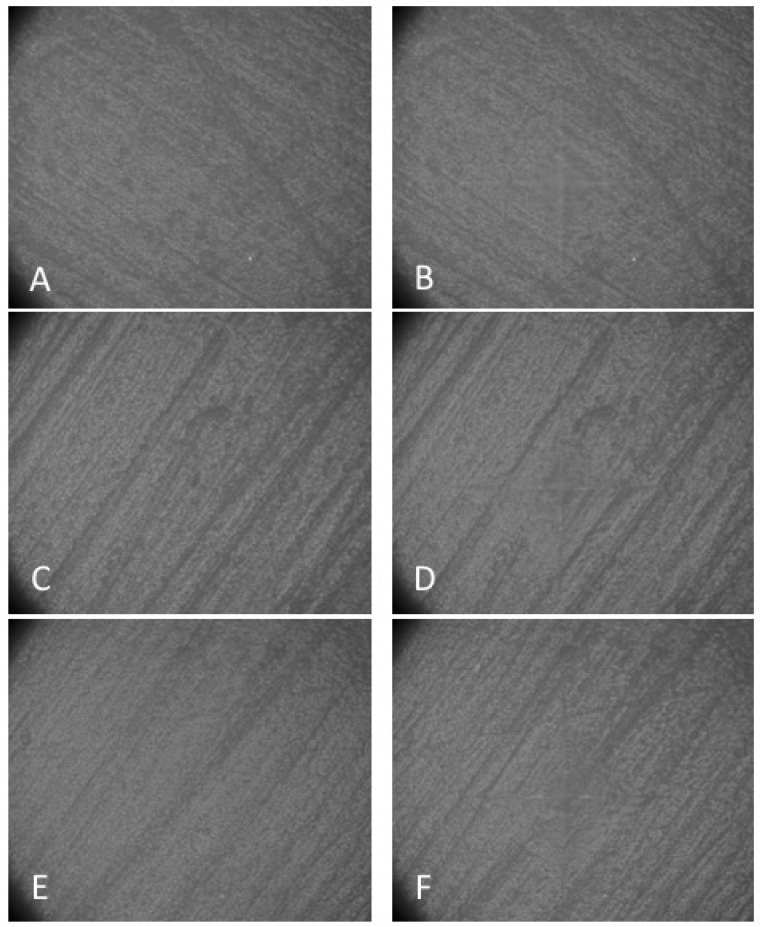
Photomicrographs of nanohybrid composite (Grandio blocs) before and after immersion in acidic drink for 7 and 28 days (40× magnification). (**A**,**B**) Before immersion; (**C**,**D**) after immersion in acidic drink for 7 days, (**E**,**F**) after immersion in acidic drink for 28 days.

**Figure 5:**
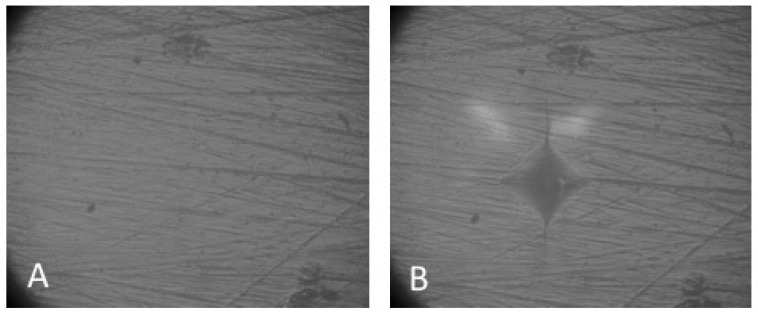

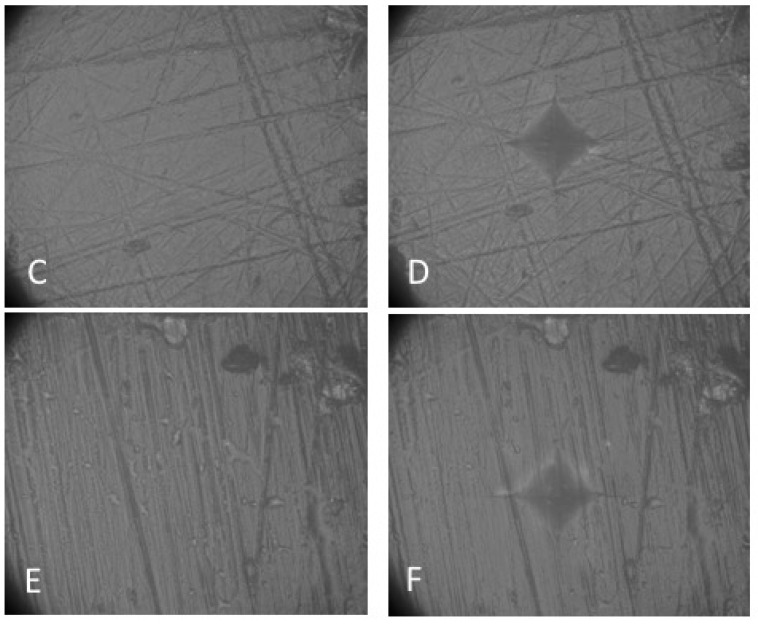
Photomicrographs of reinforced glass ceramic (VITA SUPRINITY^®^ PC) before and after immersion in acidic drink for 7 and 28 days (40× magnification). (**A**,**B**) Before immersion; (**C**,**D**) after immersion in acidic drink for 7 days, (**E**,**F**) after immersion in acidic drink for 28 days.

The purpose of the present study was to test the micro-hardness (Figure 2, Figure 3, Figure 4 and Figure 5) of four different recently introduced CAD/CAM indirect restorative materials, and the results showed significant differences among various groups.

The lowest micro-hardness values were reported with hybrid ceramic blocks (CERASMART™) (Group 1). The hybrid ceramic was previously tested for mechanical properties [24,25], showing similar indentation values (67.30 ± 2.04). Moreover, micro-hardness and color stability after exposure to an acidic substance (coffee and red wine) have been tested previously [25]. The results indicated that coffee can affect the color and micro-hardness of tested materials. This is in agreement with the results of our study.

In the present study, higher indentation values were reported with resin nano ceramic (Lava™ Ultimate) (Group 2). This material has been previously tested [17,24], including the evaluation of its mechanical properties elastic modulus, flexural strength, and micro-hardness. The reported micro-hardness (112.20 ± 10.2) values were similar (slightly higher) to the results of our report at T0. However, the materials were tested without exposure to an acidic drink, in contrast to the present study. Another report [26] evaluated the effects of simulated gastric juice on the mechanical properties of a CAD/CAM resin composite (i.e., micro-hardness). The acid scenario (immersion in an acidic solution for 6 and 24 h) did not change their micro-hardness. These results are in contrast with the present study because the exposure to an acid scenario was shorter, even if the pH solution was lower (gastric solution pH = 1.2; Coca-Cola pH = 2.52).

Another material tested in our study was the nanohybrid composite (Grandio blocs), which showed higher micro-hardness values than the other two materials previously discussed. This material has been previously tested for edge force [27] and micro-tensile bond strength [27,28], but there are no indentation studies yet. There are many studies on the effect of sports and energy drinks on the surface hardness of different restorative materials [1,29]. However, nowadays there are few studies on the effect of acidic drinks on the micro-hardness of restorative CAD/CAM materials.

In the present report, the highest micro-hardness values were reached with zirconia-reinforced lithium silicate glass-ceramic (VITA SUPRINITY^®^ PC). This glass ceramic is an innovative material, introduced in 2013, with a dual microstructure composed of lithium metasilicate (Li_2_SiO_3_) and lithium disilicate (Li_2_Si_2_O_5_) in a glass matrix containing zirconium oxide in solution [30]. In the literature, there are studies regarding the optical properties [31] and mechanical properties [32] of this material, but there are no reports on its micro-hardness.

After immersion in Coca-Cola (pH = 2.52) for 7 and 28 days, the micro-hardness of all tested materials was negatively affected. Zirconia-reinforced lithium silicate glass ceramic (VITA SUPRINITY^®^ PC) was less affected, presumably because of its high chemical stability. This is probably due to the composite component or polymeric matrix incorporated in the other three materials (hybrid ceramic, resin nano ceramic, nanohybrid composite), which appeared more affected by the acidic solution than the reinforced glass ceramic. In fact, dental composite resins have a polymeric network that can be negatively influenced by exposure to acidic beverages. It is reported that mechanical properties, in particular micro-hardness, are affected by long-term immersion in an acidic environment [33].

Dental ceramics are considered chemically inert restoration materials; however, exposure to acidic erosive agents can undermine the stability and durability of ceramics. A study [34] demonstrated that exposure to an acid scenario leads to a decrease in the micro-hardness values of ceramics because of the dissolution of the materials; elementary components such silica, aluminum, and potassium are released by the glass phase. This dissolution has also been reported with other dental materials used in conservative dentistry, such as resin composites [35] and fiber-reinforced composites [36]. In fact, the oral cavity is a complex aqueous environment, modified by everything that is introduced daily. For example, food and beverages can lower the salivary pH values. In this way, the physical and mechanical properties of restorative CAD/CAM materials can be changed [37]. The loss of mechanical characteristics, such as flexural strength, flexural modulus, and, particularly for this study, micro-hardness, is caused by factors such as saliva, acids, and bases [38].

The results obtained at T0 confirmed that CAD/CAM resin composites have greater micro-hardness characteristics than other materials tested. In general, the mechanical characteristics of ceramic-based materials are stronger than composite-containing materials [39,40,41]. However, the present report tested only micro-hardness, and this is a limitation of the study. Future research is needed in order to test other mechanical characteristics to complete the overview of the behavior of the materials tested.

The exposure to acidic drink in this study was continuous, without rinsing the specimens daily, in order to simulate long-term exposure to carbonated beverages in the oral cavity. Immersion in Coca-Cola for 24 h is comparable to the consumption of the beverage for one month, considering that it remains in the mouth while drinking [42]. In a recent study [25], immersion in the acidic drink was tested for 7 days (T1) and 28 days (T2); the second immersion protocol is comparable to more than two years of in vivo conditions.

As confirmed by other studies [43], the loss of the mean micro-hardness percentage is higher during the first 7 days of exposure (T0–T1), while during the other 21 days (T1–T2) it is lower. The cited study was performed on resin composites for direct restorations; we obtained analogous results of the mean micro-hardness percentage, although in the present study restorative CAD/CAM materials were used. Our experimental design evaluated the behavior of the materials after 7 and 28 days of acidic drink immersion, as many reports in dental literature evaluated similar time intervals [1,13,43]. It would be interesting in the future to test the same materials with more measurements at shorter time periods. These new tests could be useful to check the hardness evolution along time, thus allowing extrapolation to longer service periods [44].

Immersion in Coca-Cola was performed at room temperature (18 ± 1 °C), even if is reported that acidic drinks have a lower erosive power if they are consumed at a low temperature [45]. The present investigation did not evaluate other drinks (e.g., water, tea, and milk) because of their lower acidic concentrations compared with Coca-Cola [2,4].

The oral cavity includes many variables that cannot be simulated by in vitro conditions. Therefore, further clinical research is needed to confirm the present results.

Data availability statement: All the data are available upon request to andrea.scribante@unipv.it. 

## 5. Conclusions

- Coca-Cola has an erosive power on the surface micro-hardness of restorative CAD/CAM materials, particularly during the first 7 days of immersion.
- Ceramics have greater mechanical characteristics, particularly in the case of micro-hardness, than resin composites and are less affected by the acidic solution.

## Figures and Tables

**Table 1:** Characteristics of the materials used in the study.

Group	Material	Type	Composition	Manufactured
1	CERASMART™	Hybrid ceramic	Bis-MEPP, UDMA, DMA, silica (20 nm), barium glass (300 nm)	GC Corporation, Tokyo, Japan
2	Lava™ Ultimate	Resin nano ceramic	Bis-GMA, UDMA, Bis-EMA, TEGDMA, SiO_2_ (20 nm), ZrO_2_ (4–11 nm), aggregated ZrO_2_/SiO_2_ clusters	3M, Minnesota, USA
3	Grandio blocs	Nanohybrid composite	86% *w*/*w* inorganic filler in a polymeric matrix	VOCO GmbH, Cuxhaven, Germany
4	VITA SUPRINITY^®^ PC	Reinforced glass ceramic	56–64% SiO_2_, 15–21% Li_2_O, 8–12% ZrO_2_, <10% pigments	VITA Zahnfabrik, Bad Sackingen, Germany

**Table 2:** Micro-hardness values between different immersion protocols. (T0: baseline; T1: immersion for 7 days; T2: immersion for 28 days).

Material	T0	T1	T2	
	Mean (SD)	Mean (SD)	Mean (SD)	*p*-Value
Hybrid ceramic (CERASMART™)	68.2 (0.5) ^a^	64.8 (0.5) ^b^	62.93 (0.3) ^c^	<0.001
Resin nano ceramic (Lava™ Ultimate)	95.9 (0.2) ^a^	91.5 (0.5) ^b^	88.2 (0.4) ^c^	<0.001
Nanohybrid composite (Grandio blocs)	130.6 (0.5) ^a^	122 (0.4) ^b^	117.2 (1.7) ^c^	<0.001
Reinforced glass ceramic (VITA SUPRINITY^®^ PC)	731.4 (2.6) ^a^	717.9 (2.2) ^b^	712.5 (1.1) ^c^	<0.001

SD: standard deviation. *: Significant difference at *p* < 0.05. Superscript letters (a, b and c) have been used to indicate statistical results: Different letters among times (T0, T1 and T2) indicate significant difference in micro-hardness for the material tested.

**Table 3:** Mean percentage micro-hardness loss for the investigated materials between different immersion protocols.

Material	T0-T1	T1-T2	T0-T2
Hybrid ceramic (CERASMART™)	4.97 (1.1) ^a^	2.93 (0.8) ^a^	8.41 (1.3) ^a^
Resin nano ceramic (Lava™ Ultimate)	4.54 (1.2) ^a^	3.66 (0.9) ^a^	8.73 (1.2) ^a^
Nanohybrid composite (Grandio blocs)	6.58 (1.4) ^b^	3.93 (1.1) ^a^	11.43 (1.3) ^b^
Reinforced glass ceramic (VITA SUPRINITY^®^ PC)	1.85 (0.7) ^c^	0.75 (0.6) ^b^	2.65 (0.7) ^c^

*: Significant difference at *p* < 0.05. Superscript letters (a, b and c) have been used to indicate statistical results: Different letters among time intervals (T0-T1, T1-T2 and T0-T2) indicate significant difference in micro-hardness for the material tested.
